# Association between cognitive deficits and suicidal ideation in patients with major depressive disorder

**DOI:** 10.1038/s41598-017-12142-8

**Published:** 2017-09-14

**Authors:** Shenghong Pu, Shiori Setoyama, Takamasa Noda

**Affiliations:** 1 Integrative Brain Imaging Center, National Center Hospital, National Center of Neurology and Psychiatry, 4-1-1 Ogawa-Higashi, Kodaira, Tokyo 187-8551 Japan; 2 Division of Neuropsychiatry, Department of Brain and Neuroscience, Tottori University Faculty of Medicine, 36-1 Nishi-cho, Yonago, Tottori 683-8504 Japan; 3 Department of Psychiatry, National Center Hospital, National Center of Neurology and Psychiatry, 4-1-1 Ogawa-Higashi, Kodaira, Tokyo 187-8551 Japan

## Abstract

The role of cognitive function in suicidal ideation in patients with major depressive disorder (MDD) has not been adequately explored. This research sought to measure the relationship between suicidal ideation and cognitive function. Therefore, in this study, the association between cognitive function and suicidal ideation in patients with MDD was assessed. Cognitive function was evaluated in 233 patients with MDD using the Japanese version of the Brief Assessment of Cognition in Schizophrenia (BACS). Suicidal ideation was assessed using item 3 of the Hamilton Depression Rating Scale. Approximately 59.2% of the patients (138/233) expressed suicidal ideation. Among the BACS subtests, only the executive function scores were significantly lower in patients with MDD with than in those without (p < 0.005). In addition, the executive function, motor speed function, and composite scores correlated negatively with the severity of suicidal ideation in these patients. These results suggest that executive function, motor speed function, and global neuropsychological function are associated with suicidal ideation in patients with MDD and that the BACS neuropsychological battery is an efficient instrument for monitoring these characteristics. Moreover, specific BACS scores can potentially serve as cognitive biomarkers of suicide risk in patients with MDD.

## Introduction

Suicidal ideation is a major public health concern. Approximately one million suicides and ten million suicide attempts have been recorded worldwide each year; in 2012, suicide was the 15th leading cause of death worldwide^1, 2^. However, despite numerous studies and introduction of policies aimed at improving methods for predicting and preventing suicide, suicidal ideation and attempts have remained virtually unchanged^3, 4^. Thus, it is essential to improve our ability to predict and ultimately prevent suicide.

Indeed, approximately one-third of suicide ideators proceed to attempt suicide^5, 6^. More importantly, suicidal ideation represents an enduring vulnerability rather than a short-term crisis^7^. These findings imply that suicidal ideation likely precedes suicide and that suicidal behavior never occurs without prior contemplation. Therefore, studies investigating suicidal ideation are important for gaining an understanding of the link between suicidal ideation and the risk of suicide attempt^4, 8^.

Suicidal ideation is a common, core symptom of major depressive disorder (MDD)^9^. In fact, previous reports have suggested that 22.4% to 66% of patients with MDD experience suicidal ideation in the week prior to attempting suicide^7, 10–12^. Patients with MDD also show cognitive deficits in neuropsychological domains, such as visual and verbal memory, working memory, attention, executive function, and processing speed^13^, with impairments in executive function being the most prominent^14, 15^. In particular, impairments in cognitive control^16^, which refers to the ability to regulate one’s own thoughts and actions in order to achieve internal goals^17^, have been associated with depression-related pathology. Cognitive control is a low-level cognitive process that underlies rule learning by integrating feedback with prior knowledge of contingency and environment structure. Thus, cognitive control allows flexible adaptation of behavior to meet the current demands, particularly in the face of ambiguous, complex, and/or changing environments^18^. Several studies have suggested that impaired cognitive control abilities^16, 19^ may underlie the high suicide rate found in individuals with depression^20^.

Neurocognitive deficits appear to be a risk factor for suicidal behavior^21^ and are presumed to lead to an increased risk of suicide due to an incorrect appraisal of one’s life situation and consequently to poor decision-making^18^. Indeed, impaired neurocognitive functioning has been found in patients with a history of suicide attempts^22–27^ and in those with current suicidal ideation^28, 29^. With respect to the neuropsychological correlates of suicidal ideation in patients with MDD, Marzuk *et al*.^28^ found that patients with current suicidal ideation performed significantly worse than patients without suicidal ideation on several executive tasks, such as the Wisconsin Card Sorting Test. This led the authors to conclude that current suicidal ideation, regardless of the history of suicide attempts, is associated with impaired executive function. Recently, Gujral *et al*.^30^ reported that depressed older individuals with current suicidal ideation, with or without a history of suicide attempts, are impaired in executive function. Owing to the significant risk of suicidal ideation in patients with MDD^31, 32^, it is important to elucidate the relationship between executive function and suicidal ideation in patients with MDD.

The present study primarily aimed to investigate the relationship between cognitive function and suicidal ideation in patients with MDD in a precise manner, using the Brief Assessment of Cognition in Schizophrenia (BACS). The BACS is one of the most popular assessment batteries for determining neurocognitive function in individuals with severe psychopathology^33^. It consists of various tests that assess the following six cognitive domains: verbal memory, working memory, motor speed, verbal fluency, attention and speed of information processing, and executive function. The BACS was originally developed for patients with schizophrenia, but has also been used for those with bipolar disorder^34, 35^, and recently for those with MDD^36, 37^. Importantly, the use of this comprehensive battery consisting of highly reliable and valid tests that were selected specifically for their tolerability in patients with various psychiatric disorders, will facilitate comparisons across numerous studies. Here, we hypothesized that patients with current suicidal ideation would have greater dysfunction in specific cognitive domains (for example, executive function) than would patients without suicidal ideation; furthermore, the degree of dysfunction in cognitive domains would correlate with the severity of current suicidal ideation.

## Material and Methods

### Participants

After the study procedures had been explained, written informed consent was obtained from each participant. This study was approved by the ethics committee of the National Center of Neurology and Psychiatry (approval no. A2011-037) and the investigation was conducted in accordance with the latest version of the Declaration of Helsinki (2013).

In total, 233 patients with MDD, aged between 16 and 76 years, who were outpatients of the National Center of Neurology and Psychiatry Hospital in Tokyo, Japan, between January 2010 and December 2016, participated in the study (Table 1). Patients were diagnosed in accordance with the Structured Clinical Interview for Diagnostic and Statistical Manual of Mental Disorders, Fourth Edition, Axis I Disorders (SCID-I) by experienced psychiatrists. All of the patients with MDD were in a depressed mood state (17-item Hamilton Rating Scale for Depression [HAM-D] score >7). Among the included patients, 201 were medicated with one or more agents (antidepressants, antipsychotics, mood stabilizers, anxiolytics, and/or antiparkinsonian agents), while 32 patients were drug-free. Daily doses of all the antidepressants were converted to an equivalent dose of imipramine^38^, antipsychotics were converted to that of chlorpromazine^38^, and doses of anxiolytics/hypnotics were converted to that of diazepam^38^.

**Table 1:** Demographic and clinical characteristics of patients with MDD with and without suicidal ideation.

Demographics	MDD with suicidal ideation (n = 138) (mean ± SD)	MDD without suicidal ideation (n = 95) (mean ± SD)	Statistics	*p* value
Age (years)	39.9 ± 13.4	41.6 ± 13.4	t (df = 231) = 0.914	0.362
**Sex** ^**a**^, **n (%)**				
Male (n = 111)	56 (40.6)	55 (57.9)	X^2^ = 6.76	0.009
Female (n = 122)	82 (59.4)	40 (42.1)		
Education, years (n = 225)	14.7 ± 2.3	14.8 ± 2.2	t (df = 223) = 0.104	0.917
Age at Onset^b^ (n = 218)	32.4 ± 13.8	32.9 ± 13.3	U = 5570.5	0.743
Duration of illness (years)^b^ (n = 218)	7.5 ± 6.8	8.2 ± 6.8	U = 5312.5	0.372
**Suicidal ideation**				
HAM-D item 3^b^	1.96 ± 0.82	0.00	U = 0.000	p < 0.001
HAM-D^b^	17.6 ± 6.0	12.2 ± 4.3	U = 2915.5	p < 0.001
**Treatment** ^**a**^ **(%)**				
Medication (n = 201)	117 (84.8)	84 (88.4)	X^2^ = 0.63	0.429
Drug free (n = 32)	21 (15.2)	11 (11.6)		
Imipramine-equivalent dose (mg/day)^b^	139.5 ± 137.0	139.5 ± 137.0	U = 6136.0	0.402
Chlorpromazine-equivalent dose (mg/day)^b^	94.8 ± 212.0	69.4 ± 140.3	U = 6434.5	0.786
Diazepam-equivalent dose (mg/day)^b^	9.8 ± 11.5	9.8 ± 13.4	U = 6340.5	0.669

Note: MDD, Major Depressive Disorder; HAM-D, 17-item Hamilton Depression Rating Scale. Significant group differences are shown to the right. ^a^Chi-square test and ^b^Mann−Whitney U-test were used for testing group differences. Otherwise, t-tests were used. p < 0.05 was considered significant.

### Clinical assessments

#### Depression

Depressive symptoms were evaluated by a single experienced psychiatrist using the 17-item HAM-D.

### Suicidal ideation

Suicidal ideation was measured on a scale of 0 to 4 using the suicide item (item 3) from the HAM-D. Individuals receiving a score of 0 were considered to have “no suicidal ideation.” Scores of 1 or above were taken to indicate the presence of suicidal ideation. This cutoff value has commonly been used in previous studies^4, 8, 39^. Participants with MDD were divided into two groups according to the presence or absence of suicidal ideation^4, 8, 39^.

### Cognitive function

Cognitive function was assessed by administering the Japanese version of the BACS to each participant^33, 40, 41^. The BACS evaluation included tests, such as the List Learning Test, Digit Sequencing Task, Token Motor Task, Category Instances Test and Controlled Oral World Association Test, Symbol Coding, and Tower of London Test, which measure verbal memory, working memory, motor speed, verbal fluency, attention and speed of information processing, and executive function. The primary measure from each test of the BACS was standardized by creating z-scores, whereby the mean score of Japanese healthy controls was set to zero, and the standard deviation was set to one^41^. Composite scores were calculated by averaging the z-scores of all six subcomponents (composite score = [verbal memory z-score + working memory z-score + motor speed z-score + verbal fluency z-score + attention and speed of information processing z-score + executive functions z-score]/6), with higher scores reflecting higher cognitive function.

### Statistical analyses

Statistical analyses were performed using the SPSS 22.0 software (Tokyo, Japan). Categorical variables were compared using chi-squared tests. Clinical variables that were normally distributed were compared using *t-*tests, while Mann−Whitney *U* tests were used for clinical variables that were not normally distributed. The BACS scores were compared using *t*-tests. To examine the relationship between the BACS scores, HAM-D score, and suicidal ideation (item 3 of the HAM-D), Spearman’s correlation coefficients were calculated. In addition, to elucidate the independent contributions of the BACS scores that showed significant correlations with suicidal ideation, we performed stepwise multiple regression analyses. In these analyses, we adjusted for other potential confounding variables, such as age, sex (dummy parameterized, male = 1, female = 0), education, duration of illness, treatment (dummy parameterized, medication = 1, drug free = 0), and depressive symptoms (HAM-D score), suicidal ideation (item 3 of the HAM-D) was the dependent variable, with a probability of F for a conservative entry and removal criteria of 0.05 and 0.1, respectively. For significant findings, effect sizes are indicated using the standardized regression coefficient (β). Suicidal ideation and depression were calculated using the raw HAM-D item 3 scores and the remaining HAM-D items, respectively. Differences were considered significant at *p* < 0.05.

## Results

### Demographic and clinical characteristics of patients with MDD with and without suicidal ideation

Of the 233 participants, 138 (59.2%) expressed suicidal ideation. Table 1 summarizes the demographic and clinical characteristics of the patients; no differences in the characteristics were noted between the patients, with and without suicidal ideation, except for sex (*χ* ^2^ = 6.67, *p* = 0.009) and the HAM-D score (*U* = 2915.5, *p* < 0.001).

### Cognitive deficits in patients with MDD with and without suicidal ideation

Compared to patients with MDD without suicidal ideation, those with suicidal ideation demonstrated significantly lower BACS scores in the executive function domain (*t* = 2.905, df = 231, *p* < 0.005) (Table 2). No differences were noted between the groups with respect to the other cognitive domains or composite scores.

**Table 2:** Cognitive deficits of patients with MDD with and without suicidal ideation.

Demographics	MDD with suicidal ideation (n = 138) (mean ± SD)	MDD without suicidal ideation (n = 95) (mean ± SD)	Statistics	*p*.value
BACS				
Verbal memory	−1.179 ± 1.424	−1.011 ± 1.358	t (df = 231) = 0.898	0.370
Working memory	−0.593 ± 1.247	−0.363 ± 1.284	t (df = 231) = 1.368	0.173
Motor speed	−1.693 ± 1.565	−1.345 ± 1.536	t (df = 231) = 1.678	0.095
Verbal fluency	−0.319 ± 1.247	−0.353 ± 1.326	t (df = 231) = −0.198	0.843
Attention and speed of information processing	−1.307 ± 1.390	−1.136 ± 1.345	t (df = 231) = 0.933	0.352
Executive function	−0.613 ± 1.412	−0.099 ± 1.195	t (df = 231) = 2.905	0.004
Composite score	−1.761 ± 1.687	−1.338 ± 1.702	t (df = 231) = 1.871	0.063

Note: MDD, Major Depressive Disorder; BACS, Brief Assessment of Cognition in Schizophrenia.

### Correlations

The composite score was modestly negatively correlated with the HAM-D score (ρ = −0.20, *p* < 0.005).

The executive function and motor speed domain scores were weakly negatively correlated with the HAM-D score (ρ = −0.17, *p* < 0.01 and ρ = −0.16, *p* < 0.05, respectively).

The executive function, motor speed, and composite scores were negatively correlated with suicidal ideation (item 3 of the HAM-D) (ρ = −0.19, *p* < 0.005; ρ = −0.16, *p* < 0.05; and ρ = −0.16, *p* < 0.05, respectively). None of the other cognitive domain scores were associated with suicidal ideation (Table 3).

**Table 3:** Correlation coefficients between the cognitive ability and suicidal ideation, and depressive symptoms in patients with MDD.

	Suicidal ideation	Depressive symptoms
	HAM-D item 3	HAM-D
BACS		
Verbal memory	−0.09	−0.11
Working memory	−0.08	−0.11
Motor speed	−0.16*	−0.16*
Verbal fluency	−0.06	−0.14
Attention and speed of information processing	−0.09	−0.11
Executive function	−0.19***	−0.17**
Composite score	−0.16*	−0.20***

Note: MDD, Major Depressive Disorder; HAM-D, 17-item Hamilton Depression Rating Scale; BACS, Brief Assessment of Cognition in Schizophrenia. *p < 0.05. **p < 0.01. ***p < 0.005.

The HAM-D score was positively correlated with suicidal ideation (item 3 of the HAM-D) (ρ = 0.449, *p* < 0.001).

Additionally, we performed stepwise multiple regression analyses to elucidate the independent contributions of the BACS scores that showed significant correlations with suicidal ideation. When only male patients were considered, executive function (β = 0.219; *p* = 0.022) correlated with suicidal ideation, even after controlling for depressive symptoms (HAM-D score) (Table 4). These relationships were not significant when all patients or only data from female patients were analyzed (Table 4).

**Table 4:** Stepwise multiple regression predicting the suicidal ideation from neurocognition and depressive symptoms.

	R^2^	Adjusted R^2^	Independent Variables		Other Factors
			BACS executive function		
			β	*p*	
All patients^a^	0.206	0.203	−0.095	0.129	HAM-D score: β = 0.454, *p* < 0.001; BACS motor speed: β = −0.077, *p* = 0.217; BACS composite score: β = −0.073, *p* = 0.242
Male^b^	0.155	0.138	−0.219	0.022	HAM-D score: β = 0.285, *p* = 0.003; BACS motor speed: β = 0.000, *p* = 0.999; BACS composite score: β = −0.065, *p* = 0.555
Female^b^	0.270	0.264	0.014	0.862	HAM-D score: β = 0.520, *p* < 0.001; BACS motor speed: β = −0.096, *p* = 0.242; BACS composite score: β = −0.073, *p* = 0.380

Note: BACS, Brief Assessment of Cognition in Schizophrenia; HAM-D, 17-item Hamilton Depression Rating Scale. ^a^BACS scores (executive function, motor speed, composite score), age, sex (dummy parameterized, male = 1, female = 0), education, duration of illness, treatment (dummy parameterized, medication = 1, drug free = 0), and depressive symptoms (HAM-D score) were included in the multiple linear regression analysis. ^b^BACS scores (executive function, motor speed, composite score), age, education, duration of illness, treatment (dummy parameterized, medication = 1, drug free = 0), and depressive symptoms (HAM-D score) were included in the multiple linear regression analysis.

## Discussion

The current study found significant differences in cognitive deficit levels in the executive function domain between patients with MDD, with and without suicidal ideation. Furthermore, significant negative correlations were observed between the cognitive function levels, specifically the executive function domain, motor speed domain, and composite scores, and the scores for item 3 (suicidal ideation) of the HAM-D, in patients with MDD. Our findings suggest that the BACS executive function domain, motor speed domain, and composite scores are associated with suicidal ideation in patients with MDD and that the BACS is an efficient instrument for monitoring these characteristics. To our knowledge, this is the first study to indicate an association between the BACS neuropsychological battery scores and suicidal ideation in patients with MDD.

The present findings concur with those of previous studies suggesting that executive function is associated with suicidal ideation in patients with MDD^4, 28, 29^. Most of the existing reports on suicide risk in patients with depression have noted a close relationship between deficits in executive function and MDD. For example, a preliminary study revealed deficits in executive functions in depressed patients with suicidal behavior^24^, while another reported that poor performances on tests of executive function, attention, and memory were associated with suicidal behavior in patients with late-life depression^42^. Moreover, as mentioned earlier, Marzuk *et al*.^28^ suggested that current suicidal ideation, regardless of the history of suicide attempts, may be associated with impaired executive function. Recently, a study from our laboratory reported an interesting relationship between neural activity in the prefrontal cortex, which controls executive function, and suicidal ideation^4^. The prefrontal cortex exercises executive control of information processing and behavioral expression, including the ability to attend to and maintain information selectively, inhibit irrelevant stimuli, and evaluate and select the appropriate response^43, 44^. Hence, the presence of suicidal ideation could reflect, or be a reflection of, cognitive rigidity within the executive domain^29^.

In addition, the present study demonstrated interesting relationships between the BACS motor speed domain and composite scores and suicidal ideation. In this study, motor speed was assessed with the Token Motor Task, which specifically evaluates psychomotor speed^45, 46^. Psychomotor disturbances are a classic feature of MDD that reportedly emerge from alterations in the limbic signals, which influence emotion, volition, higher-order cognitive function, and movement^47^. Westheide *et al*.^29^ also found that only suicide attempters with current suicidal ideation, but not attempters without current suicidal ideation, displayed cognitive impairments in decision-making and motor inhibition^30, 48^. Collectively, these findings suggest that dysfunction in specific cognitive domains (e.g., executive function and motor speed) and global neuropsychological impairment (BACS composite score) may be associated with suicidal ideation in patients with MDD who are currently in a depressive state.

The present study found a significant negative relationship between executive function and suicidal ideation in men with MDD, even after removing the effects of confounders, such as the HAM-D score, by using multiple regression analyses with stepwise selection. The results of these analyses suggested that the effect of executive function on current suicidal ideation may be mediated by factors other than mood, particularly in men with MDD.

The present findings need to be interpreted within the context of the following limitations. First, this study was cross-sectional; therefore, it was difficult to verify the causal relationship between suicidal ideation and cognitive deficits. Second, the effects that cognitive deficits have on the outcome of suicidal death could not be verified. Third, suicidal ideation was examined only with item 3 of the HAM-D rather than with psychometric instruments specifically developed for examining this factor. Item 3 of the HAM-D is not a well-validated or in-depth assessment of suicidal ideation. Detailed assessment of the three hypothesized domains of suicidal ideation (i.e., cognitive, emotional, and behavioral) may have yielded more detailed results^49^. However, the present study did not include some variables that might contribute to executive function impairments, such as the patients’ suicide attempt histories^24^, due to the lack of available information about the participants. Further studies with longitudinal designs that control for diverse covariates are warranted.

The current study indicated that patients with MDD with or without suicidal ideation could be distinguished by the presence of cognitive deficits in the executive function domain. Moreover, executive function, motor speed function, and global neuropsychological impairment (BACS composite score) were each associated with the severity of suicidal ideation. However, further studies are required to verify these findings. If confirmed, our work may allow investigators to evaluate the potential of the BACS neuropsychological battery to serve as a cognitive biomarker of the suicide risk in patients with MDD.
